# Phosphorylation of 4E-BP1 in the Mammalian Brain Is Not Altered by LRRK2 Expression or Pathogenic Mutations

**DOI:** 10.1371/journal.pone.0047784

**Published:** 2012-10-17

**Authors:** Alzbeta Trancikova, Adamantios Mamais, Philip J. Webber, Klodjan Stafa, Elpida Tsika, Liliane Glauser, Andrew B. West, Rina Bandopadhyay, Darren J. Moore

**Affiliations:** 1 Brain Mind Institute, School of Life Sciences, Ecole Polytechnique Fédérale de Lausanne, Lausanne, Switzerland; 2 Reta Lila Weston Institute of Neurological Disease, University College London Institute of Neurology, London, United Kingdom; 3 Center for Neurodegeneration and Experimental Therapeutics, Department of Neurology, University of Alabama at Birmingham, Birmingham, Alabama, United States of America; University of Sheffield - MRC Centre for Developmental and Biomedical Genetics, United Kingdom

## Abstract

Mutations in the *leucine-rich repeat kinase 2* (*LRRK2*) gene are a common cause of autosomal dominant familial Parkinson's disease (PD). *LRRK2* encodes a multi-domain protein containing GTPase and kinase enzymatic domains. Disease-associated mutations in LRRK2 variably influence enzymatic activity with the common G2019S variant leading to enhanced kinase activity. Mutant LRRK2 induces neuronal toxicity through a kinase-dependent mechanism suggesting that kinase activity is important for mediating the pathogenic effects of LRRK2 mutations. A number of LRRK2 kinase substrates have been identified *in vitro* but whether they represent authentic physiological substrates in mammalian cells or tissues is not yet clear. The eukaryotic initiation factor 4E (eIF4E)-binding protein, 4E-BP1, was recently identified as a potential substrate of LRRK2 kinase activity *in vitro* and in *Drosophila* with phosphorylation occurring at Thr37 and Thr46. Here, we explore a potential interaction of LRRK2 and 4E-BP1 in mammalian cells and brain. We find that LRRK2 can weakly phosphorylate 4E-BP1 *in vitro* but LRRK2 overexpression is not able to alter endogenous 4E-BP1 phosphorylation in mammalian cells. In mammalian neurons LRRK2 and 4E-BP1 display minimal co-localization, whereas the subcellular distribution, protein complex formation and covalent post-translational modification of endogenous 4E-BP1 are not altered in the brains of LRRK2 knockout or mutant LRRK2 transgenic mice. In the brain, the phosphorylation of 4E-BP1 at Thr37 and Thr46 does not change in LRRK2 knockout or mutant LRRK2 transgenic mice, nor is 4E-BP1 phosphorylation altered in idiopathic or G2019S mutant PD brains. Collectively, our results suggest that 4E-BP1 is neither a major nor robust physiological substrate of LRRK2 in mammalian cells or brain.

## Introduction

Mutations in the *leucine-rich repeat kinase 2* (*LRRK2*, PARK8) gene cause late-onset, autosomal dominant Parkinson's disease (PD), and represent the most common cause of inherited PD [1], [2], [3]. *LRRK2* mutations are also prevalent in sporadic PD in some populations, whereas more common genetic variation in the *LRRK2* gene associates with PD in genome-wide association studies [1], [3], [4], [5]. The clinical, neurochemical and neuropathological spectrum of *LRRK2*-linked PD is largely indistinguishable from idiopathic PD [1], [6], [7], [8]. Therefore, *LRRK2* plays an important role in the development of familial and sporadic PD.

The *LRRK2* gene encodes a large multi-domain protein belonging to the ROCO protein family [9]. LRRK2 contains a Ras-of-Complex (ROC) GTPase domain and a C-terminal of ROC (COR) domain followed by a serine/threonine kinase domain with similarity to the mixed-lineage kinase family. Surrounding the central ROC-COR-kinase catalytic core region are a number of putative protein-protein interaction domains including N-terminal ankyrin and armadillo-like repeats, a leucine-rich repeat region, and a C-terminal WD40-like repeat domain. Mutations known to cause PD are clustered within the central catalytic region including the GTPase (N1437H, R1441C, R1441G and R1441H), COR (Y1699C) and kinase (G2019S and I2020T) domains [9]. Mutations alter enzymatic activities that include enhanced kinase activity (i.e. G2019S and N1437H) [10], [11], [12], reduced GTPase activity (i.e. R1441C/G/H and Y1699C) [13], [14], [15], [16] or enhanced GTP-binding (i.e. N1437H, R1441C/G/H and Y1699C) [17] of LRRK2. LRRK2 mutations have also been shown to enhance neuronal toxicity compared to the wild-type (WT) protein through a mechanism dependent on kinase and/or GTPase activity [17], [18], [19], [20]. Therefore, alterations in the enzymatic activity of LRRK2 due to pathogenic mutations are most likely important for the development of PD.

LRRK2 can act as a functional kinase *in vitro* whereby it can mediate autophosphorylation or phosphorylation of generic kinase substrates (i.e. myelin basic protein) [10], [17], [18], [21], [22], [23], [24]. The most common mutation, G2019S, is located within a DYG motif within the kinase activation domain and robustly enhances kinase activity [11]. A number of putative substrates for LRRK2 kinase activity have been identified *in vitro* including moesin [22], 4E-BP1 [25], β-tubulin [26], FoxO1 [27], MAPKK proteins [28], [29] and ArfGAP1 [30], [31], but it is unclear whether these proteins act as physiological substrates of LRRK2 in mammalian cells or tissues. 4E-BP1 is known to function as a repressor of protein translation by binding to the eukaryotic translation initiation factor, eIF4E, leading to inhibition of cap-dependent translation [32]. Phosphorylation of 4E-BP1 at Thr37 and Thr46 serves to prime subsequent phosphorylation at Ser65 and Thr70 which disrupts the interaction with eIF4E and results in the activation of protein translation [33], [34].

4E-BP1 was previously suggested to be a LRRK2 substrate with phosphorylation occurring at two specific residues, Thr37 and Thr46 [25]. Both human LRRK2 and *Drosophila* LRRK (dLRRK) mediated the phosphorylation of human 4E-BP1 or d4E-BP, respectively, *in vitro*. Silencing of dLRRK reduced whereas dLRRK overexpression enhanced d4E-BP phosphorylation at Thr37/46 in *Drosophila* [25], [35]. Furthermore, the overexpression of human LRRK2 enhanced the phosphorylation of 4E-BP1 at Thr37/46 and to a lesser extent at Thr70 in HEK-293T cells [25]. While these observations potentially suggest that 4E-BP1 is a physiological LRRK2 substrate, a recent study by Kumar and colleagues suggests that 4E-BP1 may be a relatively weak substrate of LRRK2 kinase activity *in vitro* compared to LRRK2 autophosphorylation, and they were unable to confirm the phosphorylation of 4E-BP1 by LRRK2 in cells [36].

To better define a potentially important interaction between LRRK2 and 4E-BP1, we have explored the effects of LRRK2 expression and pathogenic mutations on the phosphorylation status of 4E-BP1 in the mammalian brain using transgenic and knockout mice that are now available. Our data demonstrate that modulation of LRRK2 expression does not influence 4E-BP1 phosphorylation at Thr37 and Thr46 in mammalian cells or brain tissue. We conclude that 4E-BP1 is not a major or robust physiological substrate of LRRK2 in mammalian cells or brain.

## Results

### Phosphorylation of 4E-BP1 by LRRK2 *in vitro* but not in HEK-293T cells

**Figure 1:**
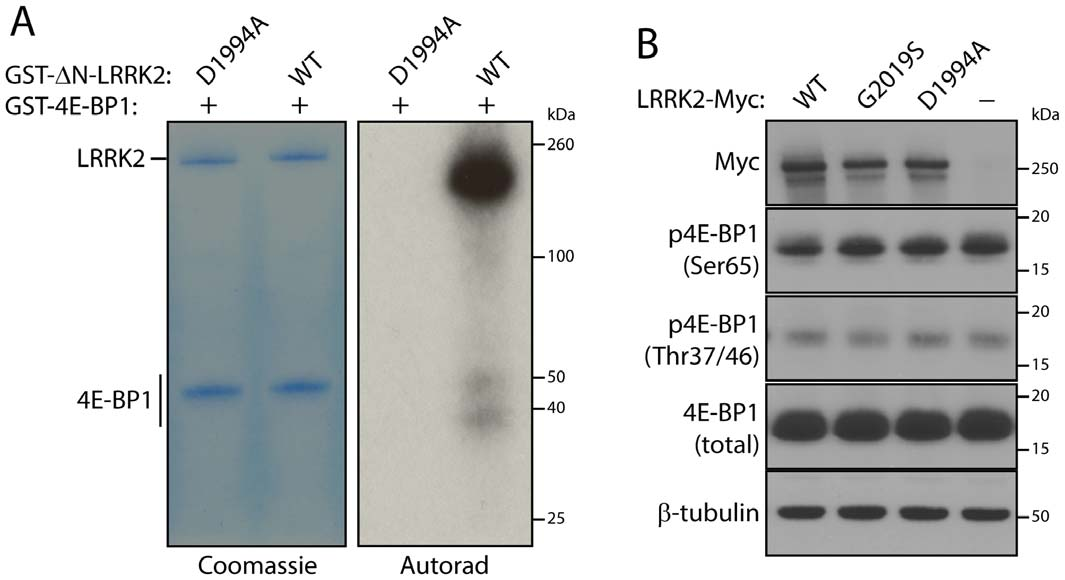
Phosphorylation of 4E-BP1 by LRRK2 *in vitro* and in mammalian cells. (**A**) *In vitro* kinase assay with [^32^P]-γ-ATP, recombinant GST-tagged human LRRK2 (ΔN, residues 970–2527) and GST-tagged human 4E-BP1. Coomassie-stained SDS-PAGE gels indicate equal loading of 4E-BP1 and LRRK2 proteins in each condition. Autoradiographs indicate the phosphorylation of 4E-BP1 by WT LRRK2 compared to kinase-inactive D1994A LRRK2. Autophosphorylation of WT LRRK2 is also detected. (**B**) Western blot analysis of endogenous 4E-BP1 phosphorylation at Thr37/Thr46 or Ser65 in HEK-293T cells transiently expressing myc-tagged human LRRK2 variants (WT, G2019S and D1994A). LRRK2 overexpression fails to alter 4E-BP1 phosphorylation. Blots are representative of duplicate experiments. Molecular mass markers are indicated in kilodaltons (kDa).

We first sought to confirm the phosphorylation of 4E-BP1 by LRRK2 *in vitro* under optimized LRRK2 activity conditions. We employed recombinant GST-tagged human LRRK2 consisting of amino acids 970-2527 together with GST-tagged human 4E-BP1 for *in vitro* kinase assays with [^32^P]-γ-ATP. Notably, the 4E-BP1 recombinant protein was highly soluble and derived from bacteria and therefore has no inherent phosphorylation modifications. We could confirm that wild-type (WT) LRRK2 modestly phosphorylates 4E-BP1 whereas kinase-inactive LRRK2 (D1994A) displays no activity (Fig. 1A). Notably, LRRK2 autophosphorylation is substantially more efficient than 4E-BP1 phosphorylation in this assay (Fig. 1A), consistent with recent reports [36]. It is possible that co-factors are required that are not present in the *in vitro* reactions, so we explored LRRK2 phosphorylation of 4E-BP1 in HEK-293T cells where 4E-BP1 is actively phosphorylated. The expression of WT or G2019S LRRK2 fails to increase 4E-BP1 phosphorylation at Thr37/46 or Ser65 relative to expression of D1994A LRRK2 or cells lacking myc-tagged LRRK2 (Fig. 1B). Collectively, these data confirm that 4E-BP1 is a rather modest substrate of LRRK2 *in vitro* and cannot influence additional phosphorylation on 4E-BP1 in HEK-293T cells even with overexpression of the kinase-hyperactive G2019S LRRK2.

### LRRK2 does not change 4E-BP1 subcellular localization or protein complexes

**Figure 2:**
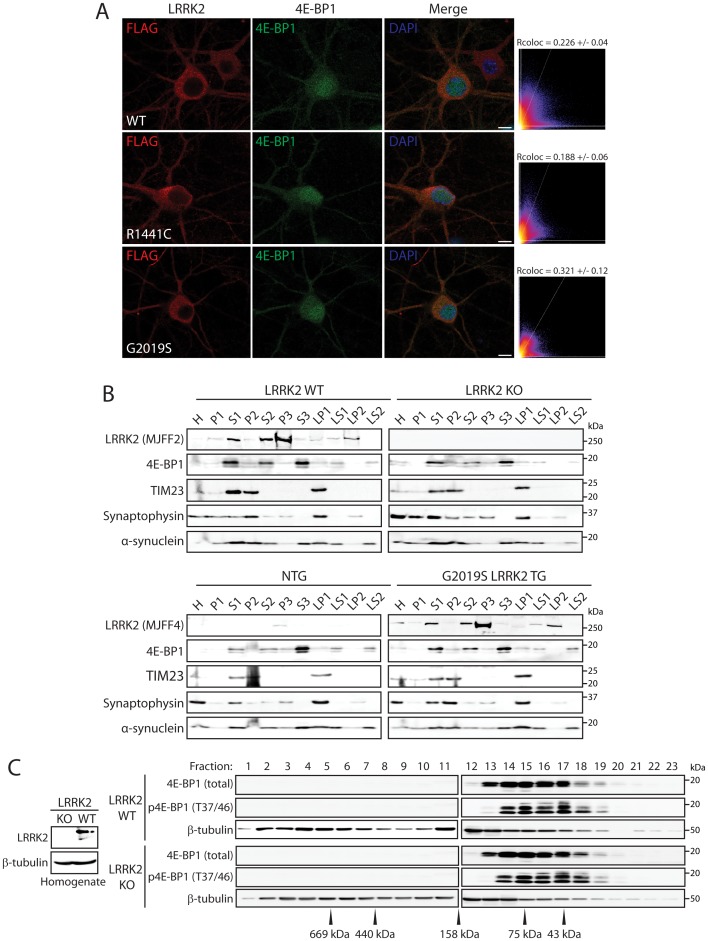
Effect of LRRK2 on 4E-BP1 subcellular localization and protein complex formation. (**A**) Confocal fluorescence microscopy reveals minimal co-localization of FLAG-tagged human LRRK2 variants and endogenous 4E-BP1 in rat primary cortical neurons. Pathogenic mutations (R1441C or G2019S) do not alter the localization of LRRK2 with 4E-BP1 compared to WT LRRK2. Cytofluorograms and co-localization coefficients (Rcoloc; mean±SEM, *n* = 5–10 neurons) reveal the extent of co-localization between LRRK2 and 4E-BP1 fluorescent signals. Confocal images are taken from single z-plane at 0.1 µm thickness. Images are representative of at least five neurons taken from duplicate experiments. Scale bar: 10 µm. (**B**) Subcellular fractionation of cerebral cortex from WT and LRRK2 KO mice, or human G2019S LRRK2 transgenic (TG) and non-transgenic (NTG) mice. 4E-BP1 is enriched in soluble cytosolic (S1, S2 and S3) fractions, and at lower levels in synaptosomal (LS1) and synaptic vesicle (LS2) cytosolic fractions. 4E-BP1 subcellular localization is not altered by LRRK2 deletion or G2019S LRRK2 expression compared to control mice. Endogenous and human LRRK2 is enriched in the microsomal (P3) fraction and at lower levels in synaptosomal membrane (LP1) and soluble cytosolic (S1 and S2) fractions. The distribution of marker proteins demonstrates the enrichment of mitochondria/heavy membranes (TIM23; P2 and LP1), synaptosomal/synaptic vesicle membranes (synaptophysin 1; P2, P3, LP1 and LP2) and synaptosomal/synaptic vesicle cytosolic (α-synuclein; LS1 and LS2). (**C**) Size-exclusion chromatography on soluble whole brain extracts from WT and LRRK2 KO mice. Sequential fractions (0.5 ml) were analyzed by Western blotting with antibodies to total or phosphorylated (Thr37/46) 4E-BP1 and β-tubulin, whereas total homogenates were probed with antibodies to LRRK2 (c41-2/MJFF2). The elution profile of 4E-BP1 is similar in WT and KO brains, whereas the elution profile of individual protein standards is indicated. Blots are representative of duplicate experiments. Molecular mass markers are indicated in kilodaltons (kDa).

Although the phosphorylation of 4E-BP1 by LRRK2 in HEK-293T cells could not be demonstrated here and also in a previous study [36], dLRRK has been reported to phosphorylate d4E-BP at Thr37/46 *in vivo* in brain extracts from *Drosophila* [25]. It is possible therefore that 4E-BP1 phosphorylation by LRRK2 occurs in a cell- or tissue-specific manner (e.g. brain tissue). To explore the relationship between LRRK2 and 4E-BP1 in the mammalian brain, we assessed the subcellular co-localization of 4E-BP1 and LRRK2 in rat primary cortical neurons. Cortical cultures were infected at DIV 6 with recombinant human adenovirus expressing full-length FLAG-tagged human LRRK2 variants (WT, R1441C or G2019S), fixed at DIV 16 and subjected to immunocytochemistry. Confocal microscopic analysis reveals limited co-localization of exogenous LRRK2 and endogenous 4E-BP1 occurring in the cytoplasm of cortical neurons whereas substantial 4E-BP1 also resides in the nucleus where LRRK2 is largely excluded (Fig. 2A). LRRK2 pathogenic mutations, R1441C and G2019S, do not influence 4E-BP1 subcellular localization or the degree of co-localization with LRRK2 in cortical neurons compared to WT LRRK2 (Fig. 2A). To isolate a possible interaction in the cytosol, we conducted subcellular fractionation of cerebral cortex tissue derived from adult LRRK2 knockout (KO) mice and their WT control littermates, or human G2019S LRRK2 transgenic and non-transgenic mice. 4E-BP1 is enriched in the soluble S1, S2 and S3 fractions and at lower levels in the synaptosomal cytosolic LS1 and synaptic vesicle cytosolic LS2 fractions but is largely excluded from the nuclear P1 fraction (Fig. 2B). In contrast, LRRK2 is enriched in the microsomal P3 fraction and at lower levels in the synaptic vesicle membrane (LP2) and soluble S1 and S2 fractions (Fig. 2B). Therefore, 4E-BP1 and LRRK2 partly co-localize in the soluble S1 and S2 fractions but otherwise exhibit distinct subcellular distribution profiles in adult mouse brain. The subcellular fractionation profile of 4E-BP1 in brain is not altered in LRRK2 KO mice or human G2019S LRRK2 transgenic mice compared to littermate control mice (Fig. 2B). To explore the impact of LRRK2 expression on 4E-BP1 protein complex formation, we conducted size-exclusion chromatography on soluble brain extracts derived from adult WT and LRRK2 KO mice. The elution profile of total and phosphorylated 4E-BP1 is similar in WT and KO mouse brain fractions without obvious differences in the levels of total or phosphorylated (Thr37/46) 4E-BP1 (Fig. 2C). Collectively, these data reveal that 4E-BP1 and LRRK2 only partly co-localize in cultured neurons and in soluble fractions of mouse brain, however, LRRK2 expression does not influence the subcellular localization, phosphorylation or protein complex formation of 4E-BP1 in the mouse brain.

### LRRK2 does not regulate the phosphorylation of 4E-BP1 at Thr37/46 in mouse brain

**Figure 3:**
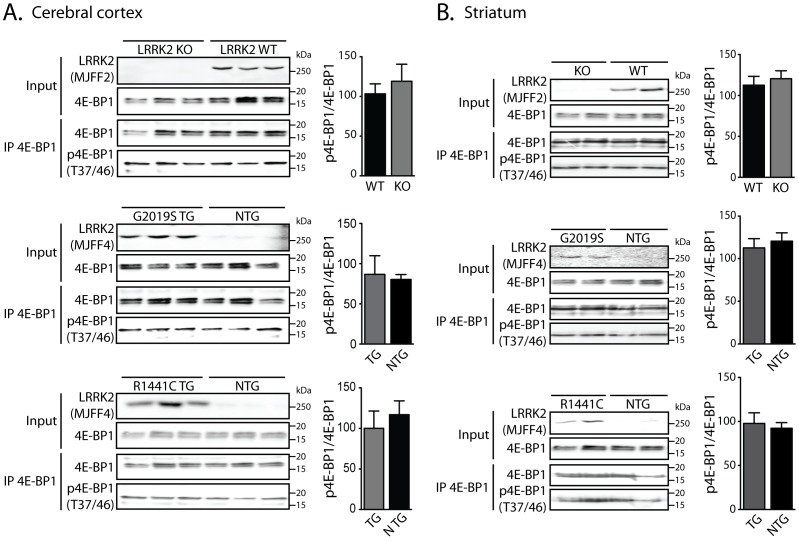
Effect of LRRK2 on 4E-BP1 phosphorylation in mouse brain. Total 4E-BP1 immunoprecipitates or input lysates from the (**A**) cerebral cortex or (**B**) striatum of WT and LRRK2 KO mice, or human LRRK2 (R1441C or G2019S) transgenic (TG) and non-transgenic (NTG) mice were analyzed by Western blot analysis with antibodies to phosphorylated (Thr37/46) and total 4E-BP1, or LRRK2 (total: c41-2/MJFF2; human-selective: c81-8/MJFF4). Densitometric analysis reveals unaltered 4E-BP1 phosphorylation by LRRK2 deletion or mutant human LRRK2 expression compared to littermate control mice. The levels of phosphorylated 4E-BP1 were normalized to total 4E-BP1 and expressed as a percent of control mice (mean±SEM, *n* = 3 mice/genotype). Molecular mass markers are indicated in kilodaltons (kDa).

To explore the impact of LRRK2 expression and pathogenic mutations on 4E-BP1 phosphorylation in mouse brain, total 4E-BP1 was immunoprecipitated from cerebral cortex extracts of WT and LRRK2 KO mice, or from human R1441C or G2019S LRRK2 transgenic mice and non-transgenic littermate control mice. 4E-BP1 immunoprecipitates were analyzed by Western blotting with antibodies recognizing total or phosphorylated (Thr37/46) 4E-BP1. The phosphorylation of 4E-BP1 at Thr37/46 is not altered by LRRK2 deletion or overexpression of mutant LRRK2 in the cerebral cortex, nor are differences in phospho-shifts noted using total 4E-BP1 antibodies (Fig. 3A). Similar observations were made in striatal extracts derived from LRRK2 KO and human LRRK2 transgenic mice compared to control mice (Fig. 3B). LRRK2 deletion in KO mice is confirmed using an antibody specific for total LRRK2 (MJFF2) whereas human LRRK2 expression in transgenic mice is confirmed using a human-selective LRRK2 antibody (MJFF4) (Fig. 3). Collectively, these data demonstrate that LRRK2 expression or pathogenic mutations (G2019S or R1441C) do not influence 4E-BP1 phosphorylation at Thr37/46 in the mouse brain.

### LRRK2 does not alter post-translational modifications of 4E-BP1 in cells or brain

**Figure 4:**
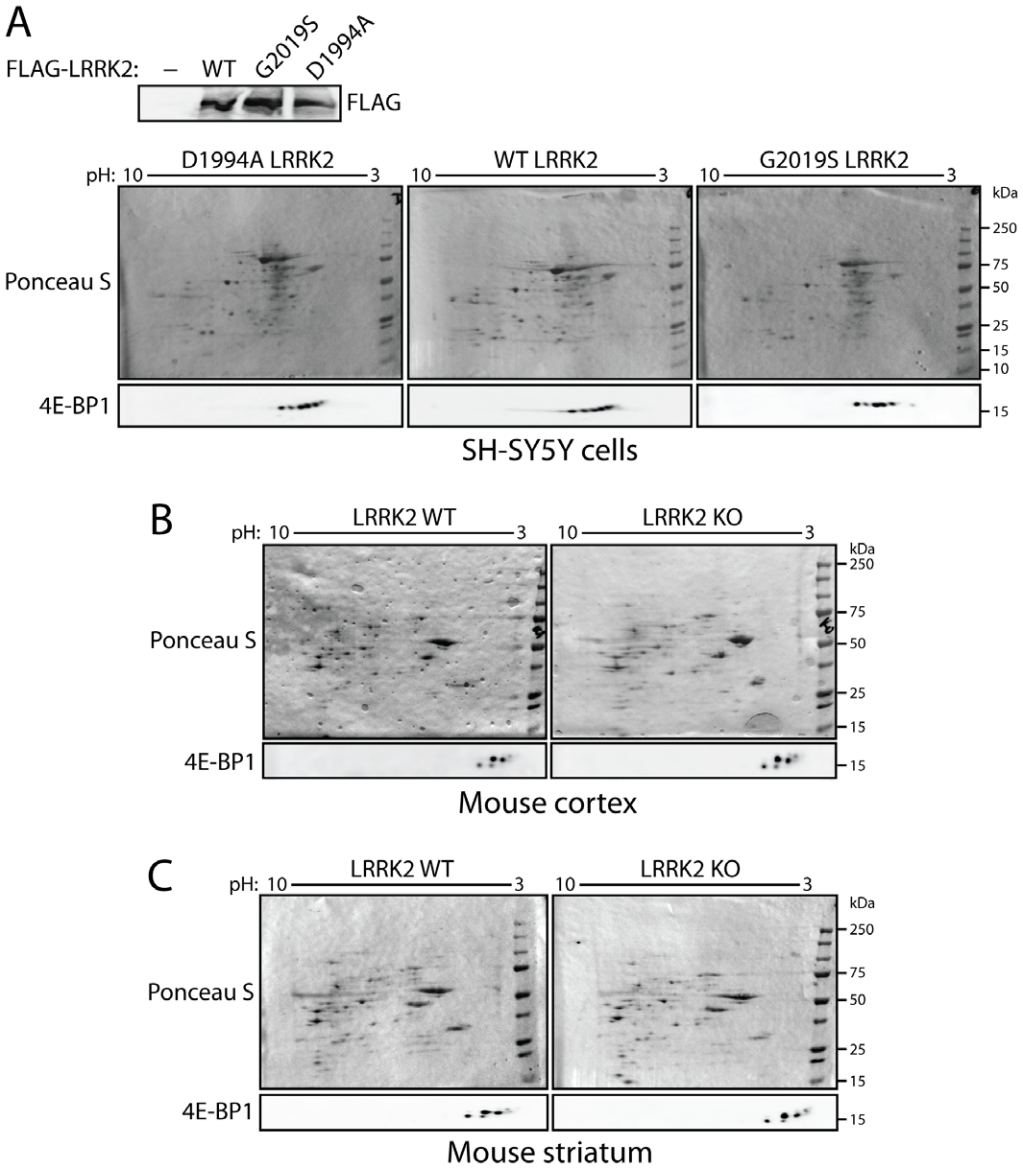
Effect of LRRK2 on 4E-BP1 post-translational modification in mammalian cells and brain. (**A**) 2D SDS-PAGE (pH 3–10 and 8–16% SDS-PAGE) analysis of SH-SY5Y cell extracts expressing FLAG-tagged human LRRK2 variants (WT, G2019S or D1994A). 2D blots were probed with 4E-BP1 antibody or stained with Ponceau S red to reveal equivalent protein loading. The 2D migration profile of 4E-BP1 is not altered by LRRK2 kinase-inactive (D1994A) or kinase-hyperactive (G2019S) mutations relative to WT LRRK2. 1D blots were probed with anti-FLAG antibody to reveal equivalent human LRRK2 levels. Blots are representative of duplicate experiments. (**B** and **C**) 2D SDS-PAGE analysis of cerebral cortex and striatum extracts derived from WT or LRRK2 KO mice with 4E-BP1 antibody or Ponceau S red as a protein loading control. The 2D profile of 4E-BP1 is not altered by LRRK2 deletion. Blots are representative of three experiments using independent mice for each genotype. Molecular mass markers are indicated in kilodaltons (kDa).

As LRRK2 fails to alter 4E-BP1 phosphorylation in mouse brain tissue, we elected to explore whether LRRK2 expression or activity could influence the post-translational modification of 4E-BP1. Such modifications could potentially reveal alternative sites of 4E-BP1 phosphorylation in addition to other covalent modifications. To assess the effects of LRRK2 kinase activity on 4E-BP1, extracts from human SH-SY5Y neural cells expressing FLAG-tagged human LRRK2 variants (WT, G2019S or D1994A) were resolved by 2D SDS-PAGE and subjected to Western blot analysis for total 4E-BP1. Endogenous 4E-BP1 is detected as ∼6 discrete acidic species of similar molecular mass in SH-SY5Y cells (Fig. 4A). However, the 2D migration pattern of 4E-BP1 is not altered by WT or G2019S LRRK2 expression compared to D1994A LRRK2 expression (Fig. 4A). We next conducted similar studies on cerebral cortex and striatal extracts derived from LRRK2 KO and WT mice. 4E-BP1 is detected as 4–5 discrete acidic species in brain tissue but this 2D migration pattern is not altered by deletion of LRRK2 (Fig. 4B and C). Collectively, these data suggest that modulating LRRK2 expression or activity in human cells or mouse brain does not alter the post-translational modification of 4E-BP1 consistent with no effect of LRRK2 on 4E-BP1 phosphorylation *in vivo*.

### Phosphorylation of 4E-BP1 at Thr37/46 in idiopathic and G2019S mutant PD brains

**Figure 5:**
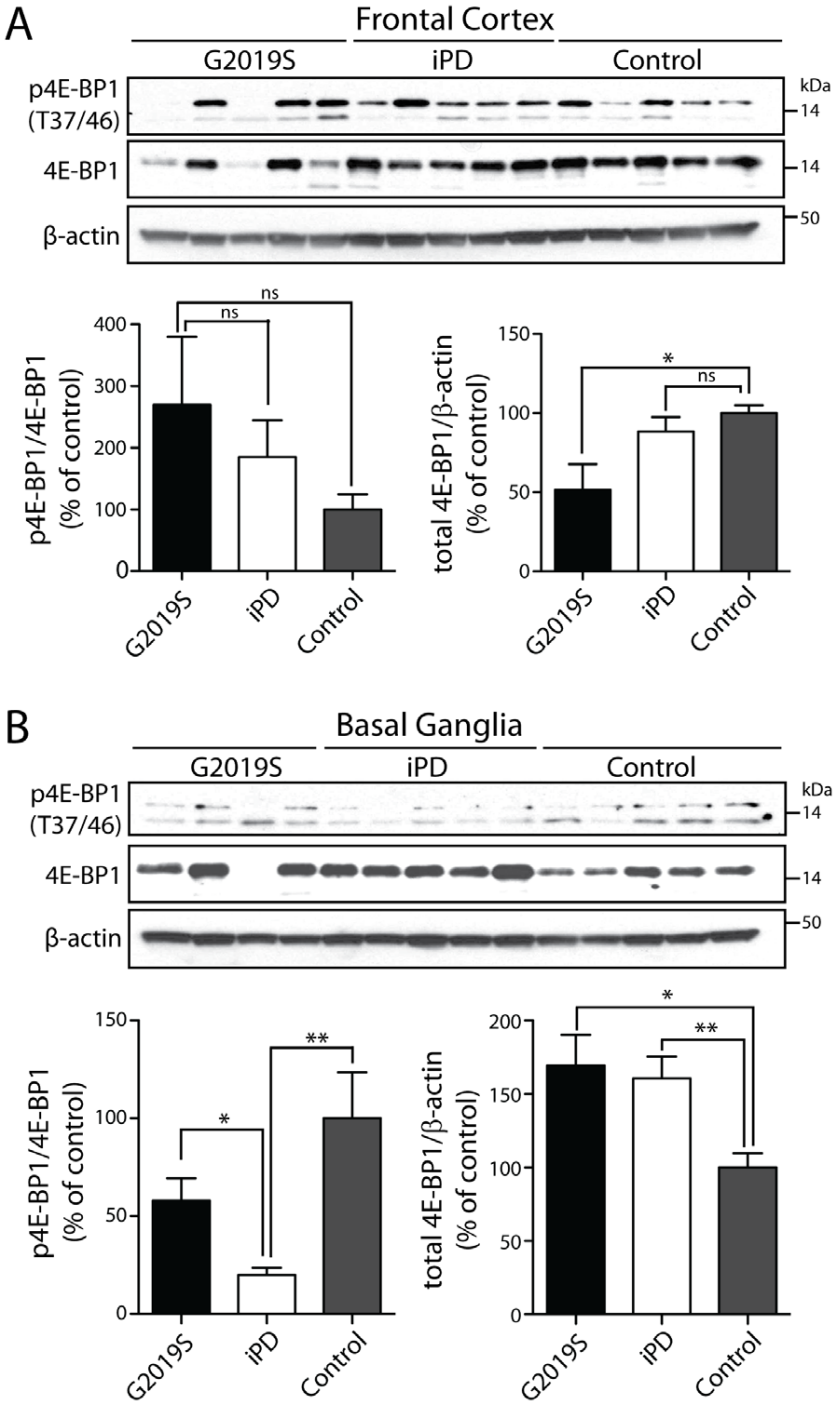
Phosphorylation of 4E-BP1 in brains of PD subjects. Western blot analysis of (**A**) frontal cortex or (**B**) basal ganglia soluble fractions from human control, idiopathic PD (iPD) and G2019S LRRK2 PD subjects with antibodies to total or phosphorylated (Thr37/46) 4E-BP1, or β-actin as a protein loading control. Molecular mass markers are indicated in kilodaltons (kDa). Densitometric analysis of 4E-BP1 phosphorylation (upper protein band) or total 4E-BP1 levels in idiopathic or G2019S PD brains compared to control brains. The levels of phosphorylated 4E-BP1 were normalized to total 4E-BP1, whereas total 4E-BP1 levels were normalized to β-actin levels, and expressed as a percent of control subjects (mean±SEM, *n* = 4–5 brains/group). For basal ganglia, G2019S subject 3 was excluded from the densitometric analysis due to a lack of detectable 4E-BP1 expression. *P<0.05 or **P<0.01 by one-way ANOVA with Newman-Keuls post-hoc analysis. *ns*, non-significant.

Since we were not able to detect LRRK2-dependent alterations in 4E-BP1 phosphorylation in human cell lines and mouse brain, we next sought to determine whether 4E-BP1 phosphorylation is altered in human brain tissue derived from PD subjects with or without *LRRK2* mutations. Soluble extracts derived from frontal cortex and basal ganglia of idiopathic or G2019S mutant PD brains and normal control brains were subjected to Western blot analysis with antibodies to total or phosphorylated (Thr37/46) 4E-BP1. In frontal cortex, we observe a significant overall reduction of total 4E-BP1 levels in G2019S mutant PD brains (in 3 out of 5 subjects) compared to control brains, whereas the level of 4E-BP1 phosphorylation is not different across brain samples (Fig. 5A). In the basal ganglia, we observe a significant increase of total 4E-BP1 levels in idiopathic (in 5 out of 5 subjects) and G2019S mutant (in 3 out of 4 subjects) PD brains compared to control brains (Fig. 5B). The levels of phosphorylated 4E-BP1 are significantly reduced in basal ganglia extracts from idiopathic PD brains compared to control brains (Fig. 5B). The detection of full-length LRRK2 in post mortem human brain extracts is problematic and has not been possible using currently available LRRK2 antibodies. The apparent alterations in total 4E-BP1 levels in G2019S and iPD brains, which for G2019S subjects is opposite between frontal cortex and basal ganglia, could potentially reflect the effects of various factors, including post mortem delay, agonal state, age, disease pathology or tissue sampling, since not all subjects reveal a consistent trend within each group as noted above. Importantly, we do not observe *increased* 4E-BP1 phosphorylation in the frontal cortex or basal ganglia of idiopathic or G2019S mutant PD brains compared to control brains suggesting that 4E-BP1 phosphorylation is not altered by *LRRK2* pathogenic mutations in the human brain.

## Discussion

The identification of physiological substrates for LRRK2 kinase activity is of major importance for understanding the pathogenic effects of disease-causing mutations, for understanding the molecular signaling pathways downstream of LRRK2 activity but upstream of LRRK2-dependent neuronal toxicity, and as potential surrogate markers of LRRK2 kinase activity *in vivo* for monitoring the actions of kinase inhibitors. To date, only a small number of putative LRRK2 substrates have been identified *in vitro* but none of these proteins have yet been confirmed as physiological or pathological substrates in mammalian cells or tissues [9]. Of the putative LRRK2 substrates identified so far, 4E-BP1 provides one of the more compelling cases since previous studies have shown that modulating LRRK2 expression in *Drosophila* or mammalian cells leads to alterations in 4E-BP1 phosphorylation [25], [35]. Despite these observations, a recent study by Kumar and colleagues was unable to confirm the phosphorylation of 4E-BP1 by LRRK2 in mammalian cells, and further demonstrated that 4E-BP1 serves as a rather weak substrate for LRRK2 *in vitro* [36]. For these reasons, we decided to extend these prior studies to explore the contribution of LRRK2 expression and pathogenic mutations to 4E-BP1 phosphorylation in the mammalian brain to begin to understand whether abnormal 4E-BP1 phosphorylation could contribute to *LRRK2*-linked PD. In the present study, we were able to replicate previous experiments showing that 4E-BP1 is weakly phosphorylated by LRRK2 *in vitro* [25], [36]. Furthermore, we could replicate recent observations from Kumar and colleagues by similarly demonstrating unaltered 4E-BP1 phosphorylation in HEK-293T cells transiently expressing LRRK2 [36].

We extended these observations to mammalian brain tissue where we could demonstrate that the deletion of LRRK2 or the expression of human LRRK2 harboring disease-causing mutations, R1441C or G2019S, failed to alter 4E-BP1 phosphorylation at Thr37 and Thr46 in the mouse brain. Furthermore, 4E-BP1 phosphorylation at these residues was not increased in brain extracts from idiopathic or G2019S mutant PD subjects compared to control subjects. Further supporting the notion that 4E-BP1 is not a physiological substrate of LRRK2 in the mammalian brain, we could show that 4E-BP1 and LRRK2 only partially co-localize and that altering LRRK2 expression or activity does not influence the subcellular localization of 4E-BP1 in neurons or the mouse brain. In addition, the deletion of LRRK2 failed to alter the formation of native 4E-BP1 protein complexes or the post-translational modification of 4E-BP1 in the mouse brain. Collectively, our data suggest that 4E-BP1 is not a major or robust substrate of LRRK2 kinase activity in the mammalian brain.

We speculate that important differences may exist between mammalian and *Drosophila* 4E-BP1 and/or LRRK2 which may account for the apparent phosphorylation of d4E-BP by dLRRK in the *Drosophila* brain [25], [35]. The nature of these potential differences are not clear at present but could reflect alterations in the function or subcellular localization between dLRRK and LRRK2 and/or d4E-BP and 4E-BP1, or a combination of these factors. For example, mammals contain two related LRRKs, LRRK1 and LRRK2, whereas *Drosophila* and other invertebrates contain a single LRRK protein indicating functional divergence in mammals. Alternatively, it is possible that 4E-BP1 phosphorylation is stimulated by stress consistent with the phosphorylation of 4E-BP1 by the stress-activated kinase p38α [36]. In this context, one could speculate that LRRK2-dependent 4E-BP1 phosphorylation may occur in aging flies due to inherent stress, as reflected by the increased sensitivity of dLRRK transgenic flies exposed to oxidative insult [25], whereas the LRRK2 knockout and transgenic mice at the ages used in this study do not develop robust brain phenotypes and might therefore be considered free of stressful stimuli [37], [38]. 4E-BP1 appears to be consistently, albeit weakly, phosphorylated by LRRK2 *in vitro* yet evidence that 4E-BP1 is a robust substrate of LRRK2 *in vivo* is lacking [25], [36]. These observations highlight the importance of verifying LRRK2 substrate phosphorylation in mammalian cells or tissues to confirm or clarify the physiological relevance of putative LRRK2 substrates. In future, we hope to apply similar analyses to other putative or novel LRRK2 substrates pending the availability of suitable phosphorylation-specific substrate antibodies. Taken together, our data allow us to conclude that 4E-BP1 is not a major or robust physiological substrate of LRRK2 kinase activity *in vivo* in the mammalian brain. We suggest that attention should now focus on other putative LRRK2 substrates to confirm or disprove their phosphorylation by LRRK2 in mammalian cells or brain tissue.

## Materials and Methods

### Ethics statement

For use of human brain tissue in this study, patients provided written informed consent and approval for the consent procedure and experiments were obtained from the NHS National Research Ethics Committee of the UK (Approval No. 02/N093). All animal experiments were approved by the SCAV (Service de la consummation et des affaires veterinaries) in the Canton de Vaud, Switzerland (Animal authorization No. 2293), and conducted in strict accordance with the European Union directive (2010/63/EU) for the care and use of laboratory animals.

### Animals

Mice and rats were maintained in a pathogen-free barrier facility and exposed to a 12 h light/dark cycle with food and water provided *ad libitum*. Pregnant female Sprague-Dawley rats were obtained from Charles River Laboratories (L'Arbresle Cedex, France) and resulting P1 rats were used for preparation of primary cortical neuronal cultures. LRRK2 knockout mice with a deletion of exon 41 were kindly provided by Drs. Giorgio Rovelli and Derya Shimshek (Novartis Pharma AG, Basel, Switzerland) [37]. Transgenic mice expressing full-length human LRRK2 (R1441C or G2019S) from a CMV-enhanced human PDGFβ promoter were described previously [38].

### Expression plasmids, antibodies and proteins

Mammalian expression plasmids containing FLAG-tagged full-length human WT and G2019S LRRK2 were kindly provided by Dr. Christopher Ross (Johns Hopkins University, Baltimore, USA) [20]. A D1994A mutation was introduced into FLAG-tagged WT LRRK2 by PCR-mediated site-directed mutagenesis using the QuickChange II XL kit (Agilent Technologies, La Jolla, CA, USA) and verified by DNA sequencing. Myc-tagged full-length human LRRK2 (WT, G2019S and D1994A) plasmids were kindly provided by Dr. Ted M. Dawson (Johns Hopkins University, Baltimore, USA) [17]. The following antibodies were employed: mouse monoclonal anti-FLAG (M2), anti-FLAG (M2)-peroxidase and anti-β-tubulin (clone TUB 2.1), and rabbit polyclonal anti-β-actin (Sigma-Aldrich, Buchs, Switzerland); rabbit monoclonal anti-LRRK2 (clones MJFF2/c41-2 and MJFF4/c81-8; Epitomics Inc., Burlingame, CA, USA); rabbit monoclonal anti-4E-BP1 (clone 53H11), anti-phospho-4E-BP1 (Thr37/46; clone 236B4) and anti-phospho-4E-BP1 (Ser65; clone 174A9) (Cell Signaling Technology, Danvers, MA); mouse monoclonal anti-c-myc-peroxidase (clone 9E10; Roche Applied Science, Switzerland); mouse monoclonal anti-TIM23 (clone 32) and α-synuclein (Syn1, clone 42) (BD Biosciences, Allschwil, Switzerland); mouse monoclonal anti-synaptophysin 1 (Synaptic Systems, Göttingen, Germany); peroxidase-conjugated anti-mouse and anti-rabbit IgG, light chain-specific secondary antibodies (Jackson ImmunoResearch, Inc., West Grove, PA, USA); anti-rabbit IgG-AlexaFluor-488 and anti-mouse IgG-AlexaFluor-633 (Invitrogen, Carlsbad, CA, USA). Recombinant GST-tagged human LRRK2 proteins (ΔN, residues 970-2527) were obtained from Invitrogen. GST-tagged full-length human 4E-BP1 was obtained from Sigma-Aldrich.

### Cell culture and transient transfection

Human SH-SY5Y neuroblastoma cells (CRL-2266; ATCC, Manassas, VA, USA [39]) and HEK-293T cells (Invitrogen) were maintained in Dulbecco's modified Eagle's media supplemented with 10% fetal bovine serum and 1x penicillin/streptomycin at 37°C in a 5% CO_2_ humidified atmosphere. For transient transfection, cells were transfected with plasmid DNAs using FuGENE HD reagent (Roche Applied Science) according to manufacturer's recommendations. Cells were routinely harvested at 48 h post-transfection for Western blot analysis.

### Primary neuronal cultures

Sprague-Dawley P1 rats were sacrificed by decapitation, whole brains were dissected, and the cerebral cortices were stereoscopically isolated and dissociated in media containing papain (20 U/ml; Sigma). Cells were grown in 35 mm dishes on glass coverslips pre-coated with mouse laminin (33 µg/ml; Invitrogen) and poly-*D*-lysine (20 ng/ml; BD Biosciences, Allschwil, Switzerland) in media consisting of Neurobasal (Invitrogen), B27 supplement (2% w/v), L-glutamine (500 µM) and penicillin/streptomycin (100 U/ml). At *days-in-vitro* (DIV) 3, cortical cultures were treated with cytosine β-*D*-arabinofuranoside (AraC, 10 µM) to inhibit glial cell division. For infection with adenoviral vectors, we used 3×10^8^ infectious units on dishes containing 3×10^5^ cells to give a MOI of 1000.

### Adenovirus production

Second generation E1, E3, E2a-deleted recombinant human serotype 5 adenoviruses (rAd) were generated as previously described [40], [41]. A modified version of the pDC511 shuttle plasmid (Microbix Biosystems Inc., Ontario, Canada) was generated containing an expression cassette consisting of a human synapsin-1 promoter, a synthetic intron, codon-optimized 3xFLAG-tagged human LRRK2 cDNA (WT, G2019S or R1441C) and a SV40 polyadenylation signal, as previously described [41], [42]. Each pDC511-LRRK2 shuttle plasmid was co-transfected with a modified FLP, frt human Ad5 genomic plasmid (pBHGfrtΔE1,3FLP; Microbix) into E2a-complementing cells (E2T) [40], and rAd production was performed according to a standard protocol [43]. Final vector stocks were purified and concentrated using the Vivapure AdenoPACK 100RT kit (Sartorius). Viral titers of purified vector stocks were determined by OD_260_ measurements and expressed as viral particles. To determine MOI units, we estimated that 1 MOI is equivalent to 40 viral particles (assuming that on average 1 out of 40 viral particles are infectious). Adenovirus stocks were stored at -80°C until further use.

### Cell fractionation and Western blotting

Transiently transfected HEK-293T or SH-SY5Y were harvested at 48 h post-transfection in 1 ml of lysis buffer (1X phosphate-buffered saline [PBS] pH 7.4, 1% Triton X-100, 1X phosphatase inhibitor cocktail 1 and 2 [Sigma-Aldrich], 1X Complete protease inhibitor cocktail [Roche Applied Sciences]). Cell lysates were rotated at 4°C for 1 h and soluble fractions were obtained by centrifugation at 17,500 *g* for 15 min at 4°C. Protein concentration of detergent-soluble fractions was determined by BCA assay (Pierce Biotechnology, Rockford, IL, USA). For western blot analysis, 50 µg of protein was resolved by SDS-PAGE, transferred to Protran nitrocellulose membrane (0.2 µm; Perkin Elmer, Schwerzenbach, Switzerland) and incubated with primary and secondary antibodies. Proteins were visualized by enhanced chemiluminescence (ECL; GE Healthcare, Glattbrugg, Switzerland) on a FujiFilm LAS-4000 Luminescent Image Analysis system. Quantitation of protein levels by densitometry was conducted on acquired images using LabImage 1D software (Kapelan Bio-Imaging Solutions, Leipzig, Germany).

### Brain fractionation and immunoprecipitation

Mice were sacrificed by cervical dislocation and decapitation and whole brains were rapidly removed and dissected and frozen on dry ice. For immunoprecipitation (IP) assays, the cerebral cortex and striatum from adult wild-type and LRRK2 KO mice (with targeted deletion of exon 41) or human R1441C or G2019S LRRK2 transgenic and non-transgenic mice was employed. Brain extracts were prepared by homogenization in TEN buffer (100 mM Tris-HCl pH 7.5; 100 mM NaCl; 10 mM EDTA; 0.5% NP-40) supplemented with 1X phosphatase inhibitor cocktail 1 and 2 (Sigma-Aldrich) and 1X Complete protease inhibitor cocktail (Roche Applied Sciences), and clarified by centrifugation at 100,000 *g* for 20 min at 4°C. The detergent-soluble supernatant fraction was quantified by BCA assay (Pierce Biotechnology). Detergent-soluble fractions (5–10 mg of protein) were incubated with 50 µl Protein G-Dynabeads (Invitrogen) pre-incubated with 5 µg of rabbit monoclonal anti-4E-BP1 antibody (clone 53H11; Cell Signaling Technology) followed by overnight rotation at 4°C. Dynabead complexes were sequentially washed twice with TEN buffer supplemented with 500 mM NaCl and twice with TEN buffer alone. Immunoprecipitates were eluted by heating at 70°C for 10 min in 2X Laemmli sample buffer (Bio-Rad AG, Reinach, Switzerland) containing 5% 2-mercaptoethanol. IP and input lysates (50 µg of protein) were resolved by SDS-PAGE, transferred to Protran nitrocellulose (0.2 µm; Perkin Elmer, Schwerzenbach, Switzerland), and subjected to Western blot analysis with anti-4E-BP1 (clone 53H11; Cell Signaling Technology), anti-phospho-4E-BP1(Thr37/46) (clone 236B4; Cell Signaling 53H11), or anti-LRRK2 antibodies (clones c41-2/MJFF2 or c81-8/MJFF4; Epitomics, Inc.) and appropriate secondary antibodies. Proteins were visualized by enhanced chemiluminescence (ECL; GE Healthcare, Glattbrugg, Switzerland) on a FujiFilm LAS-4000 Luminescent Image Analysis system. Quantitation of protein levels by densitometry was conducted on acquired images using LabImage 1D software (Kapelan Bio-Imaging Solutions, Leipzig, Germany).

### Two-dimensional SDS-PAGE

Mice were sacrificed by cervical dislocation and decapitation and whole brains were rapidly removed and dissected and frozen on dry ice. Brain extracts were resolved by 2D SDS-PAGE (1^st^ dimension: pH 3–10, non-linear gradient IEF strips; 2^nd^ dimension: 8-16% gradient SDS-PAGE) using the ZOOM IPGRunner system (Invitrogen) according to manufacturer's instructions. Briefly, brain proteins (150 µg) were rehydrated in 160 µl of rehydration buffer (8 M Urea, 2% CHAPS, 0.5% Carrier Ampholytes (Invitrogen), 0.002% Bromphenol Blue) and loaded on ZOOM IPG Strips (pH 3–10, non-linear gradient) in the ZOOM IPGRunner system for 1 h at room temperature. Proteins were first separated using isoelectric focusing (step 1: 200 V/70 Vh; step 2: 430 V/120 Vh; step 3: 750 V/200 Vh; step 4: 200 V/1650 Vh), re-equilibrated with DTT-equilibration buffer (75 mM Tri-HCl pH 8.8, 6 M Urea, 30% glycerol, 2% SDS, 0.002% Bromphenol Blue and 125 mM DTT) for 10 min at room temperature and then with alkylating solution (75 mM Tri-HCl pH 8.8, 6 M Urea, 30% glycerol, 2% SDS, 0.002% Bromphenol Blue and 125 mM iodoacetamide) for 10 min at room temperature. Proteins were resolved in the second dimension by SDS-PAGE using 8-16% gradient gels (Invitrogen). Following 2D SDS-PAGE, proteins were either transferred to nitrocellulose for Western blot analysis with anti-4E-BP1 antibody or gels were sequentially stained with ProQ Diamond fluorescent stain (532/560 nm ex/em; Invitrogen) and Coomassie colloidal blue (G250; Bio-rad) and images were captured on a GE Typhoon 9400 Imager. For 2D SDS-PAGE analysis of SH-SY5SY cells, cells transiently transfected with FLAG-tagged human LRRK2 (WT, G2019S or D1994A) plasmids were harvested at 48 h post-transfection, lysed and cell extracts (200 µg protein) were subjected to 2D SDS-PAGE as described above.

### Subcellular fractionation of mouse brain

Mice were sacrificed by cervical dislocation and decapitation and whole brains were rapidly removed and dissected and frozen on dry ice. Subcellular fractionation was conducted as described previously [44], [45], [46] using cerebral cortex tissue from adult wild-type and LRRK2 KO mice or human R1441C or G2019S LRRK2 transgenic and non-transgenic mice. Briefly, mouse brain homogenates were subjected to centrifugation at 800 *g* for 10 min at 4°C to obtain pellet nuclear/whole cell (P1) and soluble cytosolic (S1) fractions. S1 fractions were centrifuged at 9,200 *g* for 15 min at 4°C to obtain heavy membrane (P2) and soluble cytosolic (S2) fractions. The P2 fraction was further solublized and centrifuged at 25,000 *g* for 20 min at 4°C to enrich synaptosomal membranes (LP1) and synaptosomal cytosolic (LS1) fractions. The LS1 fraction was further fractionated by ultracentrifugation at 165,000 *g* for 2 h at 4°C to produce synaptic vesicle-enriched (LP2) and cytosolic (LS2) fractions. To enrich light membranes/microsomes (P3), the S2 fraction was subjected to ultracentrifugation at 165,000 *g* for 2 h at 4°C. Protein concentrations were determined by BCA assay (Pierce Biotechnology) and equal quantities of each fraction were assessed by Western blotting with specific antibodies labeling mitochondria (TIM23; P2 and LP1), synaptosomes/synaptic vesicles (synaptophysin 1; P2, P3, LP1 and LP2), and synaptosomal/synaptic vesicle cytosolic (α-synuclein; LS1 and LS2) subcellular compartments.

### Size-exclusion chromatography of mouse brain

Size-exclusion chromatography was performed at 4°C using an Akta-FPLC system (Amersham Biosciences). Mice were sacrificed by cervical dislocation and decapitation and whole brains were rapidly removed. Whole brains from adult wild-type or LRRK2 KO mice were homogenized on ice for 30 min in lysis buffer (0.1% Triton X-100 in 1X PBS containing 1X Complete protease inhibitor cocktail [Roche Applied Sciences]), briefly centrifuged, and cleared lysates were injected for FPLC. Gel filtration was conducted using a Superdex 200 10/300 GL column (Amersham Biosciences) equilibrated with lysis buffer at 0.4 ml/min. Column void volume was 8 ml, and elution volumes of standards were 9 ml for thyroglobulin (669 kDa), 10.5 ml for ferritin (440 kDa), 12.5 ml for aldolase (158 kDa), 15.5 ml for conalbumin (75 kDa), and 16.5 ml for ovalbumin (43 kDa). Fractions (0.5 ml) were analyzed by SDS-PAGE and Western blotting with anti-4E-BP1, anti-phospho-4E-BP1 (Thr37/46) and β-tubulin antibodies.

### Human brain tissue

Human tissue for these studies was obtained from the archive at Queen Square Brain Bank (QSBB). These include 4 G2019S subjects, 5 idiopathic PD and 5 control brain subjects. Frontal cortex tissue was obtained for a fifth G2019S subject from Sun Health Research Institute, USA. The details of these human subjects are listed in Table 1. Written informed consent was obtained from all patients and approval for this study was obtained from the NHS National Research Ethics Committee of the UK. The 4 *G2019S* PD subjects from the QSBB brain bank were classified neuropathologically as the limbic subtype for Lewy body pathology according to McKeith consensus criteria for the classification of DLBs [47]. In this limbic subtype, Lewy bodies are present in brainstem and substantia nigra, and are also prominently present in the limbic regions of the cortex i.e. amygdala, transentorhinal and cingulate regions, but very few Lewy bodies are detected in the frontal, temporal and parietal cortices. The fifth *G2019S* subject, from Sun Health, also harbored limbic subtype Lewy body pathology. The iPD subjects chosen were matched for pathology with the G2019S subjects, while the controls had no signs of any significant neuropathology and did not suffer from any neurological disease. Flash-frozen tissue was obtained from the basal ganglia and frontal cortex of these subjects.

**Table 1:** Clinical details of human brain tissue.

Subject	Gender	Age (yrs)	PMD (h)	Pathology
G2019S 1	F	80	44.4	Limbic
G2019S 2	F	81	15	Limbic
G2019S 3	F	84	32.2	Limbic
G2019S 4	F	72	24.55	Limbic
G2019S 5	M	85	1.66	Limbic
iPD 1	F	69	52.5	Limbic
iPD 2	M	70	61.2	Limbic
iPD 3	F	87	47.45	Limbic
iPD 4	M	75	48	Limbic
iPD 5	F	88	11.3	Limbic
Control 1	F	85	37	N/A
Control 2	M	93	112	N/A
Control 3	F	91	98.5	N/A
Control 4	M	87	36	N/A
Control 5	F	68	41.5	N/A

*Abbreviations*: iPD, idiopathic Parkinson's disease; Limbic, limbic

subtype of Lewy body pathology according to McKeith consensus

criteria for the classification of DLB; N/A, non-applicable; PMD,

post mortem delay; yrs, years.

### Fractionation of human brain tissue

10% (w/v) homogenates were prepared from 1 g tissue from basal ganglia and frontal cortex regions in homogenization buffer (20 mM Tris-HCl pH 7.4, 150 mM NaCl, 1X Complete protease inhibitor cocktail [Roche Applied Sciences] and 1X phosphatase inhibitor cocktail [Roche Applied Sciences]) with the aid of a mechanical homogenizer, and cleared by centrifugation at 1,000 *g* for 5 min at 4°C. Protein concentrations of cleared homogenates were calculated by BCA assay (Pierce Biotechnology). Thirty µg of protein were resolved on 18% Bis-Tris gels (Invitrogen) using MOPS buffer and transferred onto PVDF membranes. Blots were probed with anti-4E-BP1 and anti-phospho-4E-BP1 (Thr37/46) (Cell Signaling Technology), or β-actin (Sigma-Aldrich) antibodies, and appropriate peroxidase-conjugated secondary antibodies. Enhanced chemiluminescence (Pierce) images were captured onto X-Omat films (Kodak). Quantitation of protein levels by densitometry was conducted on scanned images using LabImage 1D software (Kapelan Bio-Imaging Solutions). For quantitation of phospho-4E-BP1 levels, the upper protein band corresponding to 4E-BP1 was used for densitometry.

### Immunocytochemistry and confocal microscopy

For co-localization of LRRK2 and 4E-BP1, rat primary cortical cultures were infected with adenoviral vectors expressing FLAG-tagged human LRRK2 variants (WT, R1441C or G2019S) at DIV 6, fixed at DIV 16 with 4% paraformaldehyde (PFA), and subjected to immunocytochemistry with mouse anti-FLAG-(M2) antibody and rabbit anti-4E-BP1 antibody followed by anti-mouse IgG-AlexaFluor-633 and anti-rabbit IgG-AlexaFluor-488 antibodies (Invitrogen). Fluorescent images were acquired using a Zeiss LSM 700 inverted confocal microscope (Carl Zeiss AG, Feldbach, Switzerland) with a Plan-Apochromat 63x/1.40 oil objective in x, y and z planes and analyzed using NIH Image J software. Images were subjected to deconvolution using HuygensPro software (Scientific Volume Imaging, Hilversum, Netherlands). Representative images are taken from a single z-plane at a thickness of 0.1 µm.

### *In vitro* radioactive kinase assays

Recombinant GST-tagged human LRRK2 protein (Δ970-2527; WT or D1994A, Invitrogen) was incubated with recombinant GST-tagged human 4E-BP1 (Sigma-Aldrich) in kinase assay buffer (20 mM Tris pH 7.4, 5 mM EGTA and 20 mM β-glycerol phosphate in 1X PBS). Reactions were initiated by addition of activation buffer to final concentrations that includes 0.1 mM [^32^P]-γ-ATP (0.2 µCi/reaction) and 20 mM MgCl_2_ and incubation at 30°C with shaking for 30 min. Reactions were terminated by placing the tubes on ice and proteins were resolved on SDS-PAGE gels and exposed to phospho-imager screens to detect ^32^P incorporation followed by staining with Coomassie colloidal blue.

### Statistical analysis

Data were analyzed by two-tailed, unpaired Student's *t*-test for pair-wise comparisons, or by one-way ANOVA with Newman-Keuls post-hoc analysis for comparison of multiple data groups, as indicated. *P*<0.05 was considered significant.
